# Symptomatic Treatment of Influenza

**Published:** 1895-05

**Authors:** 


### Selections and Abstracts.

### SYMPTOMATIC TREATMENT OF INFLUENZA.

Dr. Claus, editor of *La Flandre Medicale*, has recently written an exhaustive article on the symptoms of influenza, in which he points out that it is an error to assume that nervous individuals are most susceptible to the neuralgic pains of influenza. He recognizes the existence of a relationship between migraine and arthritis, one of the reasons given being that the antirheumatics—at the head of which he places salicylate of sodium—are equally good antineuralgics.

After reviewing the majority of new remedies which have been recommended for the treatment of the symptoms of influenza, he expresses the opinion that among these the salicylate of soda has proved the most seviceable. It is conceded that this drug has a number of disagreeable after-effects, which frequently form an obstacle to its employment; notwithstanding this, so long as no substitute was at hand, the physician was restricted to the use of this preparation.

Recently a substitute has been found in salophen, the molecule of which contains more than one-half of salicylic acid. Inasmuch as salophen is not decomposed until it reaches the intestines, it is therefore free from the gastric disturbances which are produced by the salicylate, while it acts as effectively as the latter. For these reasons Claus employed salophen with especially good success in the migraine of arthritic origin.

In twenty cases of influenza attended with variable neuralgic pains (sciatica, intercostal neuralgia, cephalalgia, and other symptoms), remarkable amelioration was at once noted after the administration of 1.0 to 2.0 grammes of salophen. In the majority of cases recovery ensued within the course of two days. The very favorable effects of this preparation, which stamps it as an antipyretic and antiarthritic *par excellence*, gives it a leading position among specific remedies recommended for the treatment of the symptoms of influenza; for salophen embodies the advantages of the salicylate of soda, without possessing the disadvantages of the latter. As regards dosage, 0.5 gramme was administered and repeated every two hours until relief of the pain had been effected.

### ANOTHER NEW ANTISEPTIC.

It is said in recent therapeutic reports that the salicylate of cadmium has a more energetic action than other salts of this metal. It gives good results in the treatment of purulent ophthalmia, in vascular engorgement of the cornea, as an astringent in mucous discharges, against syphilides, etc. Chemically-pure salicylate of cadmium is a white salt, occurring in splenddid tabular crystals, with plain face and rounded sides. It has a sweetish taste at first, then styptic. It melts above 300°, dissolves in 24 parts of water at 100°, in 68 parts at 23°, and in 90 parts at zero. It is soluble in alcohol and ether, more readily in warm; very soluble in hot glycerin, but is insoluble in chloroform and benzine.

The salt has the formula $(C_6H_4OHCOO)_2Cd$, and has 24 parts per 100 of metallic cadmium. It may be prepared either by acting on hydrated oxide or carbonate of cadmium with salicylic acid, or by precipitating salicylate of barium with sulphate of cadmium; the latter method is more complicated and the product is not so pure.—*Journal of the American Medical Association.*

